# Kangaroo mother care: EN-BIRTH multi-country validation study

**DOI:** 10.1186/s12884-020-03423-8

**Published:** 2021-03-26

**Authors:** Nahya Salim, Josephine Shabani, Kimberly Peven, Qazi Sadeq-ur Rahman, Ashish KC, Donat Shamba, Harriet Ruysen, Ahmed Ehsanur Rahman, Naresh KC, Namala Mkopi, Sojib Bin Zaman, Kizito Shirima, Shafiqul Ameen, Stefanie Kong, Omkar Basnet, Karim Manji, Theopista John Kabuteni, Helen Brotherton, Sarah G. Moxon, Agbessi Amouzou, Tedbabe Degefie Hailegebriel, Louise T. Day, Joy E. Lawn, EN-BIRTH Study Group, Qazi Sadeq-ur Rahman, Ahmed Ehsanur Rahman, Tazeen Tahsina, Sojib Bin Zaman, Shafiqul Ameen, Tanvir Hossain, Abu Bakkar Siddique, Aniqa Tasnim Hossain, Tapas Mazumder, Jasmin Khan, Taqbir Us Samad Talha, Rajib Haider, Md. Hafizur Rahman, Anisuddin Ahmed, Shams El Arifeen, Omkar Basnet, Avinash K. Sunny, Nishant Thakur, Regina Gurung, Anjani Kumar Jha, Bijay Jha, Ram Chandra Bastola, Rajendra Paudel, Asmita Paudel, Ashish KC, Nahya Salim, Donat Shamba, Josephine Shabani, Kizito Shirima, Meena Narcis Tarimo, Godfrey Mbaruku, Honorati Masanja, Louise T. Day, Harriet Ruysen, Kimberly Peven, Vladimir Sergeevich Gordeev, Georgia R. Gore-Langton, Dorothy Boggs, Stefanie Kong, Angela Baschieri, Simon Cousens, Joy E. Lawn

**Affiliations:** 1 Department of Paediatrics and Child Health, Muhimbili University of Health and Allied Sciences (MUHAS), Dar es Salaam, Tanzania; 2 Department of Health Systems, Impact Evaluation and Policy, Ifakara Health Institute (IHI), Dar es Salaam, Tanzania; 3 Centre for Maternal, Adolescent, Reproductive & Child Health (MARCH), London School of Hygiene & Tropical Medicine, London, UK; 4 Florence Nightingale Faculty of Nursing, Midwifery & Palliative Care, Kings College London, London, UK; 5 Maternal and Child Health Division, International Centre for Diarrhoeal Disease Research, Bangladesh (iccdr,b), Dhaka, Bangladesh; 6 Department of Women’s and Children’s Health, Uppsala University, Uppsala, Sweden; 7 Ministry of Health, Department of Health Services, Kathmandu, Nepal; 8 Department of Paediatrics and Child Health, Muhimbili National Hospital, Dar Es Salaam, Tanzania; 9 Golden Community, Kathmandu, Nepal; 10 Department of Maternal, Newborn, Child, Adolescent Health and Aging, World Health Organization (WHO), Dar Es Salaam, Tanzania; 11 Department of International Health, Johns Hopkins Bloomberg School of Public Health, Baltimore, MD USA; 12 UNICEF, New York, NY USA

**Keywords:** Birth, Maternal, Newborn, Coverage, Validity, Survey, Hospital records, Health management systems, Kangaroo mother care, Preterm

## Abstract

**Background:**

Kangaroo mother care (KMC) reduces mortality among stable neonates ≤2000 g. Lack of data tracking coverage and quality of KMC in both surveys and routine information systems impedes scale-up. This paper evaluates KMC measurement as part of the *Every Newborn* Birth Indicators Research Tracking in Hospitals (EN-BIRTH) study.

**Methods:**

The EN-BIRTH observational mixed-methods study was conducted in five hospitals in Bangladesh, Nepal and Tanzania from 2017 to 2018. Clinical observers collected time-stamped data as gold standard for mother-baby pairs in KMC wards/corners. To assess accuracy, we compared routine register-recorded and women’s exit survey-reported coverage to observed data, using different recommended denominator options (≤2000 g and ≤ 2499 g). We analysed gaps in quality of provision and experience of KMC. In the Tanzanian hospitals, we assessed daily skin-to-skin duration/dose and feeding frequency. Qualitative data were collected from health workers and data collectors regarding barriers and enablers to routine register design, filling and use.

**Results:**

Among 840 mother-baby pairs, compared to observed 100% coverage, both exit-survey reported (99.9%) and register-recorded coverage (92.9%) were highly valid measures with high sensitivity. KMC specific registers outperformed general registers. Enablers to register recording included perceptions of data usefulness, while barriers included duplication of data elements and overburdened health workers. Gaps in KMC quality were identified for position components including wearing a hat. In Temeke Tanzania, 10.6% of babies received daily KMC skin-to-skin duration/dose of ≥20 h and a further 75.3% received 12–19 h. Regular feeding ≥8 times/day was observed for 36.5% babies in Temeke Tanzania and 14.6% in Muhimbili Tanzania. Cup-feeding was the predominant assisted feeding method. Family support during admission was variable, grandmothers co-provided KMC more often in Bangladesh. No facility arrangements for other family members were reported by 45% of women at exit survey.

**Conclusions:**

Routine hospital KMC register data have potential to track coverage from hospital KMC wards/corners. Women accurately reported KMC at exit survey and evaluation for population-based surveys could be considered. Measurement of content, quality and experience of KMC need consensus on definitions. Prioritising further KMC measurement research is important so that high quality data can be used to accelerate scale-up of high impact care for the most vulnerable.

**Supplementary Information:**

The online version contains supplementary material available at 10.1186/s12884-020-03423-8.

## Key findings

**Table:** 

**What is known and what is new about this study?** • Scaling up kangaroo mother care (KMC) has been slow despite the strong evidence base that KMC improves survival for stable babies ≤2000 g weight. Improving data to track coverage is vital to end preventable preterm deaths, the leading cause of under-five mortality. • EN-BIRTH was a large multi-country observational study to assess validity of KMC coverage measurement (*n* = 840 mother-baby pairs) in exit-survey and routine registers. We observed content and quality of KMC and conducted interviews with health workers and data collectors to explore barriers and enablers to routine register recording.	
**Survey – what did we find and what does it mean?** • Women’s exit survey report after admission to KMC ward/corner had high sensitivity, the first validity testing for measurement.	
**Register – what did we find and what does it mean?** • We found that KMC coverage had high sensitivity in specific KMC registers. Despite the time load for multiple register filling, health workers were motivated if they saw data being used. • KMC coverage measured from KMC specific registers was more accurate than from general registers. • Routine measurement of KMC provided in other wards and for babies re-admitted to KMC wards was not assessed in our study and will be key to consider in the future. • Unnecessary duplication of KMC data elements in multiple documents needs to be streamlined to reduce burden on nurses.	
**Gap analysis for quality of care and measurement** • Observation showed coverage of KMC was not a good proxy for receiving high-quality KMC. • Gaps in quality of care were identified even for initial observation of all KMC position components and baby wearing a hat. • Detailed analyses were conducted in the two Tanzanian hospitals and found large gaps in optimal KMC daily duration/dose and feeding. Focus on supporting care providers for KMC continuity needs to be prioritised to realise the potential of this intervention. • Arrangements for families to support mother-baby pairs during admission was not always available.	
**What next and research gaps?** • Register data for babies admitted to KMC wards have potential for aggregation in routine health information systems (HMIS) to track coverage. More research is needed to assess data flow and quality at different levels of HMIS, including how to capture KMC provided in other newborn wards. • Further research is needed to explore if KMC can still be accurately reported at the typical 2–5 year population-based survey intervals by women who provided or did not provide KMC, and if sample size in household surveys is feasible to capture babies with birthweight ≤2000 g. • Measuring quality of KMC provision and experience of care is less likely to be feasible in routine information systems and further research is needed to identify the best approach. This may include special studies or perhaps routinely tracking selected specific components (e.g. wearing a hat).	

## Background

Annually an estimated 14.9 million preterm babies are born, and prematurity complications are the leading direct cause of death of children under 5 years old [1, 2]. Low- and middle-income countries (LMICs) have high preterm birth rates, yet hospital care for small and sick newborns is characterized by inadequate staffing and ill-equipped or non-existent neonatal care units [3].

Kangaroo mother care (KMC) is recommended by the World Health Organization (WHO) as the standard of care for clinically stable newborns ≤2000 grammes (g) birthweight [4]. There is evidence that KMC contributes to 40% reduction in neonatal mortality compared to conventional neonatal care [5]. KMC is defined as prolonged skin-to-skin contact between baby and mother/other caregiver, with frequent and exclusive breastmilk feeding and close follow-up after early discharge from hospital [5]. Mechanisms of effect for KMC include thermal support, protection from infection, appropriate stimulation and maximising a nurturing environment. Despite strong evidence and potential for major impact, KMC scale-up globally remains slow [6–10].

A global target for newborn survival was first set by the *Every Newborn* Action Plan (ENAP), agreed by all United Nations member states and taken up as Sustainable Development Goal 3.2 [11]. An ambitious ENAP measurement improvement roadmap selected KMC coverage as a priority indicator [12]. Coverage indicators measure the proportion of individuals receiving care (numerator) among those who need that care (denominator). As KMC includes several components, the challenge for a KMC ***numerator*** is deciding which components to measure. The ***denominator*** includes a clear birthweight cut-off at ≤2000 g, although birthweight accuracy is challenging. Additionally, the “clinical stability” component of the definition is subject to interpretation [4]. Previous reports have described the complexity involved in defining indicators to measure the coverage of KMC [12–15].

Quality of care measurement requires more than “contact” coverage indicators, and “content” coverage measures are needed. WHO quality of care framework defines quality dimensions as provision and experience of care [16]. There is currently no consensus on components of high quality KMC but components of provision of KMC position, daily duration/dose of KMC and feeding frequency and KMC supportive environment are important to consider for measurement. Descriptive analyses suggest longer daily duration of KMC is more beneficial, based on sub-analyses of mortality trials using ≥20 h of skin-to-skin contact duration per day [5]. The challenges of meeting this ideal, especially in busy KMC units with limited beds, is reflected in an observational study in Uganda; newborns only had a mean daily duration of 3 h in KMC position during the week after birth [17]. In addition to KMC position, supporting breastmilk feeding is required for impact. Preterm newborns do not have a fully developed suck reflex so they require assisted feeding support: breast milk expression with cup/spoon/nasogastric tube feeding (NGT). Frequency of feeding is individually tailored, dependent on the baby’s weight and other clinical factors, but needs to be a minimum of every 3 h.

KMC coverage measurement is further complicated as KMC is not a one-off intervention, but a process happening over days and weeks: initiation, continuation during admission in the facility and thereafter in the community with close follow-up [12, 14, 18]. KMC initiation depends on clinical stability, whether immediately after birth or several days/weeks later. Given that neonatal mortality peaks in the first few days after birth, late initiation reduces impact [5] and several ongoing trials are investigating early KMC for unstable babies [19–21]. Another KMC measurement evidence gap is for the supportive environment, including vital close family support for the continuous intervention of KMC [15].

Data for coverage of maternal and newborn health care in LMIC is mainly from population-based household surveys such as The Demographic and Health Surveys (DHS) Program and Multiple Indicator Cluster Surveys (MICS) [22]. KMC coverage is currently not captured in these household surveys and validation research has not been conducted. As KMC is recommended to be initiated in health facilities, improving routine Health Management Information Systems (HMIS) measurement is especially relevant, since ~ 80% of global births now take place in facilities [23]. Consensus was reached at a technical meeting that KMC ward/corner admission was an appropriate “contact” coverage point and KMC indicator validity testing for “content” coverage was prioritised [24] .

The *Every Newborn* Birth Indicators Research Tracking in Hospitals (EN-BIRTH) study aimed to validate selected newborn and maternal indicators for tracking of coverage and quality of care in surveys and routine facility data [18]. The detailed analysis of the EN-BIRTH KMC dataset is the topic of this paper.

## Objectives

This paper is part of a supplement based on the EN-BIRTH multi-country validation study, ‘*Informing measurement of coverage and quality of maternal & newborn care*’, and focuses on facility KMC with four objectives:

1. **Determine NUMERATOR accuracy/validity:** for survey-reported and register-recorded KMC coverage indicator measurement compared to observational data.
2. **Compare DENOMINATOR options for KMC coverage:** including target population ≤ 2000 g (true denominator for WHO recommendation) and all low birthweight babies ≤2499 g.
3. **Analyse GAPS in coverage and quality of KMC** among admissions to KMC ward/corner: right KMC position components, daily KMC duration/dose and feeding frequency to determine how coverage gaps vary depending on the measures used.
4. **Evaluate BARRIERS and ENABLERS** to routine register recording for KMC, regarding register design, filling and use.

## Methods

### Study design, study settings and study population

**Fig. 1:**
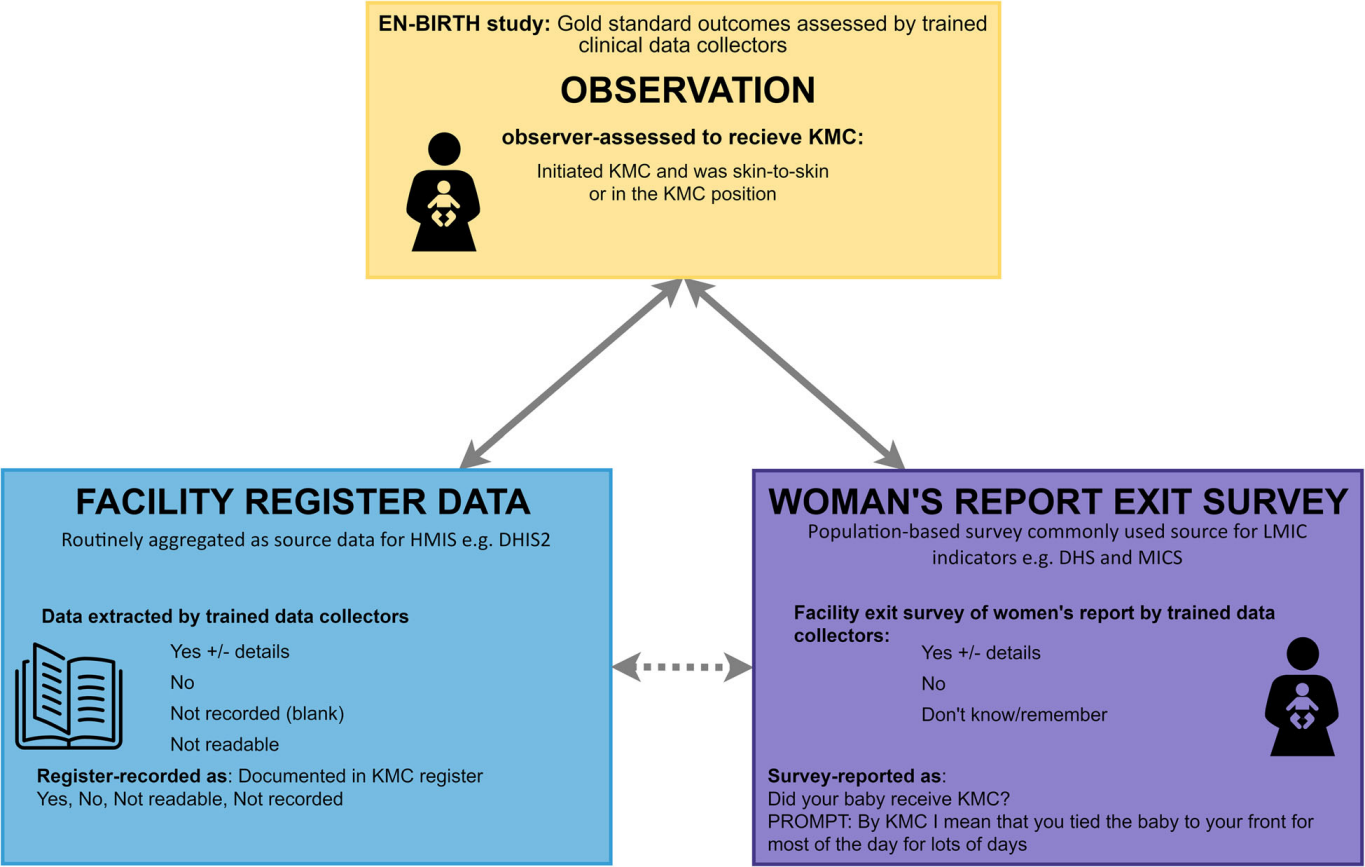
KMC validation design, EN-BIRTH study

The EN-BIRTH study was a mixed-methods observational study comparing data from clinical observers (considered the gold standard) to women’s exit survey-reported and register-recorded coverage (Fig. 1). Detailed information regarding the research protocol, methods and analysis have been published separately [18, 25]. Data were collected between June 2017–July 2018 in five public comprehensive emergency obstetric and newborn care (CEmONC) hospitals in three high mortality burden countries: Maternal and Child Health Training Institute (MCHTI), Azimpur and Kushtia General Hospital in Bangladesh (BD); Pokhara Academy Health Sciences in Nepal (NP); Temeke Regional Hospital and Muhimbili National Referral Hospital in Tanzania (TZ). Study participants for this analysis were consenting women with babies receiving routine KMC after admission to KMC wards/corners including inborn babies (born in the study hospitals) and outborns (born elsewhere). Stata version 14 was used for all quantitative analyses [26]. Results are reported in accordance with STROBE statement checklists for cross-sectional studies (Additional file 1).

### Objective 1: Numerator accuracy/validity

Research clinical observers worked in shifts covering 24 h per day. Observation was performed without interacting with the mother-baby pair. Time-stamped observation data were collected on components of KMC care. The observer collected the initial observation data as soon as possible after admission to KMC ward/corner. Admission weight was collected from individual case notes. Regular follow-up point observations for KMC position, and feeding were hourly in KMC wards in Tanzania and every 12 h in KMC corners in Bangladesh and Nepal. Women were interviewed after discharge before exit from hospital with close-ended questions regarding KMC. Researchers extracted individual mother-baby KMC data from routine hospital registers. Register designs were described and summarised. Data were collected using a custom-built android tablet-based app developed in such a way that interviewer and register extractor data collectors could not access clinical observation data, however, data were linked at individual level. Metadata for observation, survey and register are shown in Additional file 2.

Definitions of KMC coverage during admission to the KMC ward/corner are shown in Table 1. To assess accuracy at population-level (in hospital), we independently calculated and compared observed, exit survey-reported and register-recorded KMC coverage for all admitted mother-baby pairs admitted to KMC ward/corner (Fig. 1). Individual-level validity “diagnostic test” methods were calculated using two-way tables, excluding missing pairwise data. Where column totals were ≥ 10 counts, we calculated sensitivity, specificity, negative predictive value, positive predictive value, area under the curve, and inflation factor; otherwise we present percent agreement [27]. All calculations were stratified by hospital and with 95% confidence intervals (assuming a binomial distribution and using Stata's proportion and metaprop commands). We calculated I^2^ and τ^2^ to assess heterogeneity between hospitals and combined hospital-specific results using random effects meta-analysis approach.

**Table 1:** Definition of terms for KMC sample and measurement, EN-BIRTH study

KMC measurement component		EN-BIRTH study sample	Description
**KMC contact**	Total eligible population “Contact with services” (A)	Point observation - initial KMC observation	Mother-baby pairs admitted to KMC ward/corner, initial observation
**KMC continuity**		Point observation - KMC position point	Regular direct clinical observation, hourly in Tanzanian sites, 12 hourly in Bangladesh and Nepal
		Point observation - KMC feeding point	Regular direct clinical observation, hourly in Tanzanian sites, 12 hourly in Bangladesh and Nepal
**KMC coverage**	KMC position/skin-to-skin (B)	Observation KMC initiation and point observation KMC position, register-record data extraction and exit-survey report	KMC upright/vertical position and/or skin-to-skin care from any point observation during admission to discharge
**KMC content/quality**	Wearing hat (C)	Observation KMC initiation	Baby wearing hat 24 h
	KMC five position components (D)	Observation KMC initiation	1. Upright (vertical) position 2. Skin-to-skin – newborn with caregiver’s chest 3. Legs flexed in a ‘frog position’ 4. Cheek of newborn in contact with caregiver’s chest 5. Fixed firmly to caregiver’s chest (with cloth or wrap)
	KMC daily dose (E)	KMC baby days with ≥20 position point observations	Hours of per 24 h using point observation as proxy for 1 hour of KMC.
	KMC regular feeding (F)	KMC baby days with ≥8 feeding point observations	Feeds per 24 h using point observation as proxy for one feed.
	KMC supportive environment	Point observation KMC position and exit-survey report	Caregiver - mother or other family member Arrangement Pre-discharge counselling

(A) (B) (C) (D) (E) (F) refer to columns in Fig. 4

To determine reliability of the observational data, we calculated inter-rater Cohen’s Kappa coefficients for the same 5% sample observed by both supervisors and data collectors. We also calculated Kappa coefficients for a 5% sample of double-extracted study register data.

### Objective 2: Denominator options for KMC coverage

We explored KMC coverage measurement using two possible newborn admission weight denominator options: 1) ≤2000 g as the true denominator for ‘newborns in need of KMC’ as recommended by WHO, 2) ≤2499 g as some national programmes recommend KMC for all low birthweight (LBW) babies. We used KMC ward/corner admission weight as outborns may not be weighed at birth and inborns may be transferred after stabilisation for days/weeks on other neonatal wards.

### Objective 3: Gaps in coverage and quality of KMC and measurement

We measured coverage of key recommended components of KMC as markers of high-quality content KMC, to determine how coverage gaps vary depending on the measure used.

#### Dimension: provision of care – components of KMC

We designed a gap analysis figure for (A) total eligible population of newborns admitted to KMC. Among those receiving any KMC (upright/vertical and/or skin-to-skin) (B), the KMC components used as markers of high quality KMC or “right” position content evaluated were:

##### All five hospitals (initial observation)

(C) wearing a hat, (D) five position components: 1. Upright/vertical 2. Skin-to-skin contact on caregiver’s chest 3. Legs flexed in a ‘frog position’ 4. Cheek in contact with caregiver’s chest 5. Fixed with cloth/wrap to caregiver’s chest.

##### Two Tanzanian hospitals (observed and survey-report)

We further selected the subset of KMC baby days with sufficient point observations in each 24 h period to capture KMC quality for: daily duration (hereafter called KMC daily dose) ≥20 position point observations and ≥ 8 feeding observations. We calculated: (E) KMC skin-to-skin daily dose ≥20 h/day (assuming each point observation was a proxy for 1 hour of KMC), 12–19 h and < 12 h/day [5] (F) regular feeding ≥8 times/day.

#### Dimension: experience of care - supportive KMC environment

To assess components of quality of experience of care, at each point observation we calculated the proportion of KMC given by the mother alone or by another family member’s help. We asked women to report reasons for not doing KMC, grouping them as mother-related and baby-related. At exit-survey, we asked whether there were practical arrangements for family members to be involved during KMC admission and if pre-discharge counselling had been received.

### Objective 4: Barriers and enablers to routine register recording

We evaluated KMC register documentation issues as part of the wider barriers and enablers objective in the EN-BIRTH study. Two tools were designed: a) semi-structured in-depth interview (IDI) guide and b) semi-structured focus group discussion (FGD) guide, both informed by the Performance of Routine Information System Management (PRISM) conceptual framework [28]. We interviewed two purposively sampled groups of respondents: hospital health workers involved in KMC register recording and EN-BIRTH study data collectors, sampling until saturation was reached. Qualitative data were coded using pre-identified codes based on PRISM using NVivo 12 for data management. Our analysis was based on applying the same methodology as an associated EN-BIRTH paper exploring barriers and enablers to routine labour ward register recording [18]. We identified emerging themes for KMC register recording across all five hospitals by the three register process categories 1) Design 2) Filling and 3) Perceived utility.

## Results

**Fig. 2:**
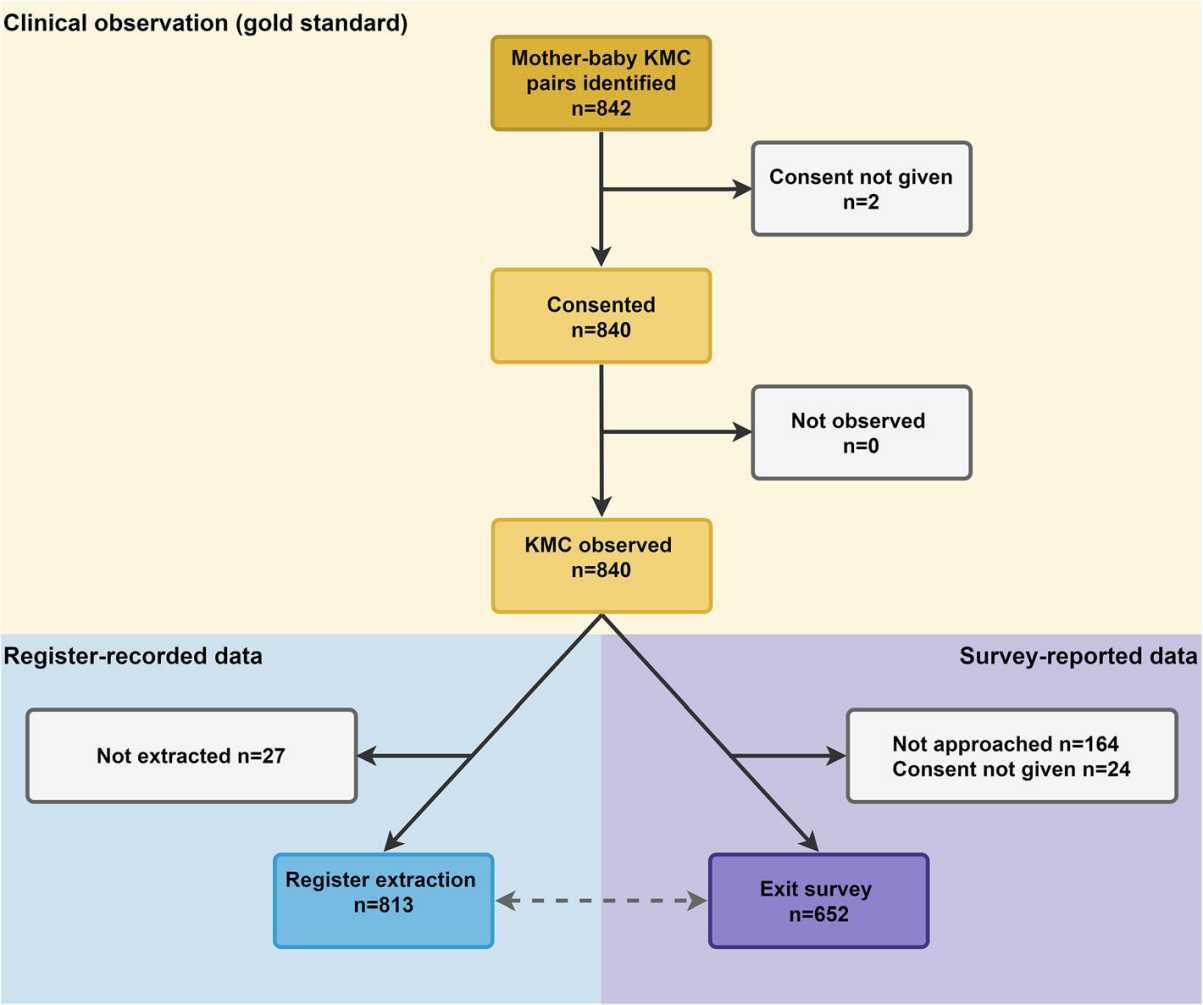
Flow diagram for KMC cases, EN-BIRTH study (*n* = 840)

Among 840 KMC mother-baby pairs observed, 77.6% of women completed exit surveys and 96.7% of register data were extracted (Fig. 2). Just over half of the KMC pairs were from the two Tanzanian hospitals. Most women (92.5%) had completed primary education, 15.9% were adolescents ≤19 years and 24.4% of babies were born by caesarean section (Additional file 3). Admission weight were available for 98% babies, mean weight was lowest at Muhimbili TZ, 1238 g, and ranging 1570-1742 g in other hopsital. 55.5% of newborns were female, and 11% were outborn. 14.4% had missing gestational age, with the highest in Temeke TZ at 30.4% (Table 2). Average age at admission to KMC ward/corner was 14.8 days in Muhimbili and between 2.9–8.1 days in the other sites. Average length of stay was 7 days, with 21.2% admitted for > 15 days. Mean discharge weight was 1629 g, although 23.6% were missing. Pre-discharge mortality was only 1.1% (Table 3).

**Table 2:** Characteristics of babies admitted to KMC ward/corners, EN-BIRTH study (*n* = 840)

Characteristics	Bangladesh		Nepal	Tanzania		Total
	Azimpur Tertiary	Kushtia District	Pokhara Regional	Temeke Regional	Muhimbili National	
	n(%)	n(%)	n(%)	n(%)	n(%)	n(%)
**Total**	**27**	**136**	**203**	**224**	**250**	**840**
**Sex of the baby**						
Male	8 (29.3)	67 (49.3)	95 (46.8)	108 (48.2)	101 (40.4)	379 (45.1)
Female	19 (70.4)	69 (50.7)	108 (53.2)	114 (50.9)	148 (59.2)	458 (54.5)
Ambiguous	0 (0.0)	0 (0.0)	0 (0.0)	1 (0.4)	0 (0.0)	1 (0.1)
Missing	0 (0.0)	0 (0.0)	0 (0.0)	1 (0.4)	1 (0.4)	2 (0.2)
**Place of delivery**						
Inborn	24 (88.9)	104 (76.5)	172 (84.7)	205 (91.5)	244 (97.6)	749 (89.2)
Outborn	3 (11.1)	32 (23.5)	31 (15.3)	19 (8.5)	6 (2.4)	91 (10.8)
**Gestational age (completed weeks)**						
< 28 (extreme preterm)	0 (0.0)	1 (0.7)	2 (0.9)	5 (2.3)	13 (5.2)	21 (2.5)
28–31 (very preterm)	2 (7.4)	24 (17.7)	12 (5.9)	42 (18.8)	125 (50.0)	205 (24.4)
32–36 (moderate/late preterm)	11 (40.0)	84 (61.8)	61 (30.1)	79 (35.3)	92 (36.8)	327 (38.9)
> 37–40	13 (48.2)	26 (19.1)	81 (39.9)	26 (11.6)	8 (3.2)	154 (18.3)
> 40	0 (0.0)	0 (0.0)	8 (3.9)	4 (1.8)	0 (0.0)	12 (1.4)
Don’t know	1 (3.7)	1 (0.7)	39 (19.2)	68 (30.4)	12 (4.8)	121 (14.4)
**Admission weight (g)**						
500–999 g	0 (0.0)	1 (0.7)	3 (1.5)	0 (0.0)	37 (14.8)	41 (4.9)
1000-1499 g	3 (11.1)	30 (22.1)	27 (13.3)	68 (30.4)	166 (66.4)	294 (35.0)
1500–1999 g	19 (70.4)	89 (65.4)	96 (47.3)	147 (65.6)	43 (17.2)	394 (46.9)
2000–2499 g	1 (3.7)	14 (10.3)	74 (36.5)	5 (2.2)	0 (0.0)	94 (11.2)
2500–4999 g	0 (0.0)	1 (0.7)	0 (0.0)	0 (0.0)	0 (0.0)	1 (0.1)
Not recorded/missing	4 (16.0)	1 (0.7)	3 (1.6)	4 (1.8)	4 (1.6)	16 (1.9)
**Weight KMC indicated (WHO)**						
≤ 2000 g	23 (85.2)	129 (94.9)	198 (97.5)	219 (97.8)	246 (98.4)	815 (97.0)
**Mean admission weight (g)**	1726	1642	1742	1570	1238	1529

Further details in Additional file 3

**Table 3:** KMC babies admitted and discharge characteristics, EN-BIRTH study (*n* = 840)

Characteristics	Bangladesh		Nepal	Tanzania		Total
	Azimpur Tertiary	Kushtia District	Pokhara Regional	Temeke Regional	Muhimbili National	
	n(%)	n(%)	n(%)	n(%)	n(%)	n(%)
**Total**	**27**	**136**	**203**	**224**	**250**	**840**
**Age of baby at admission**						
0–1 days	8 (29.6)	11 (8.1)	130 (64)	31 (13.8)	0 (0.0)	180 (21.4)
2–6 days	13 (48.1)	71 (52.2)	46 (22.7)	153 (68.3)	41 (16.4)	324 (38.6)
7–28 days	6 (22.2)	50 (36.8)	25 (12.3)	37 (16.5)	183 (73.2)	301 (35.8)
29- < 60 days	0 (0.0)	4 (2.9)	2 (1.0)	2 (0.9)	25 (10)	33 (3.9)
Missing	0 (0.0)	0 (0.0)	0 (0.0)	1 (0.4)	1 (0.4)	2 (0.2)
Mean age during admission	4.6	8.1	2.9	4.7	14.8	7.8
**Length of stay (from admission to discharge, days)**						
0–7 days	15 (55.6)	133 (90.8)	184 (90.6)	163 (72.8)	64 (25.6)	559 (66.6)
8–14 days	8(29.6)	0 (0.0)	4 (2.0)	34 (15.2)	57 (22.8)	103 (12.3)
15–21 days	4 (14.8)	0 (0.0)	0 (0.0)	12 (5.4)	47 (18.8)	63 (7.5)
22–28 days	0 (0.0)	0 (0.0)	0 (0.0)	2 (0.9)	38 (15.2)	40 (4.8)
29–55 days	0 (0.0)	0 (0.0)	0 (0.0)	1 (0.5)	35 (14.0)	36 (4.3)
Missing	0 (0.0)	3 (2.2)	15 (7.4)	12 (5.4)	9 (3.6)	39 (4.6)
Mean Length of stay	7.1	1.8	1.5	5.2	16.1	7.1
**Discharge weight (g)**						
500–999 g	0 (0.0)	0 (0.0)	4 (2.1)	0 (0.0)	1 (0.4)	5 (0.6)
1000–1999 g	18 (72.0)	89 (66.9)	69 (36.5)	197 (89.6)	183 (74.4)	556 (68.4)
2000–2499 g	5 (20.0)	8 (6.0)	30 (16.9)	9 (4.1)	5 (2.0)	57 (7.0)
2500–2599 g	0 (0.0)	1 (0.8)	0 (0.0)	0 (0.0)	1 (0.4)	2 (0.3)
Not readable	0 (0.0)	0 (0.0)	0 (0.0)	0 (0.0)	1 (0.4)	1 (0.1)
Not recorded/missing	2 (8.0)	35 (26.3)	86 (45.5)	14 (6.4)	55 (22.4)	192 (23.6)
Mean discharge weight	1875	1589	1600	1666	1596	1629
**Baby’s condition at discharge**						
Alive	27 (100)	133 (97.8)	192 (94.6)	213 (95.1)	241 (96.4)	806 (96)
Neonatal Death	0 (0.0)	0 (0.0)	3 (1.5)	4 (1.8)	2 (0.8)	9 (1.1)
Missing	0 (0.0)	3 (2.2)	8 (3.9)	7 (3.1)	7 (2.8)	25 (3.0)

Further details in Additional file 3

Standardised KMC registers were used in the hospitals in Bangladesh and Tanzania, but KMC was recorded in a non-specific column in the sick newborn register in Pokhara NP (Additional file 4). Inter-rater reliability for gold standard observation was high/substantial, except in Pokhara NP (Additional file 5).

### Objective 1: Numerator accuracy/validity

**Fig. 3:**
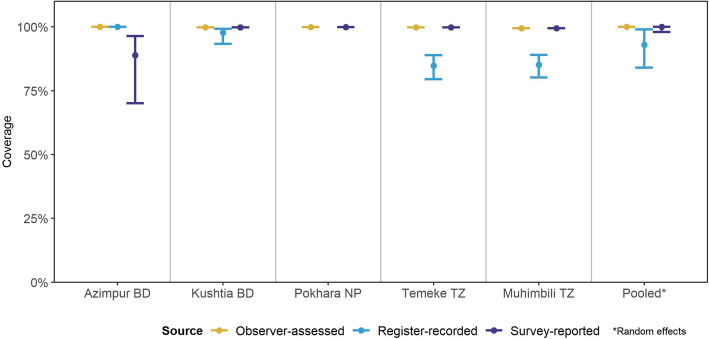
Coverage rates for KMC as measured by observation, register-record and survey-report, EN-BIRTH study (*n* = 840). Only KMC-specific register results shown and pooled for consistency. BD = Bangladesh, NP=Nepal, TZ = Tanzania

Compared to 100% observed KMC coverage (vertical/upright position and/or skin-to-skin), exit survey-reported coverage was accurate at 99.9%. Register-recorded coverage was 92.9% from standardised KMC registers, more accurate in Bangladesh hospitals, 97.8–100%, compared to Tanzanian hospitals 84.8–85.2% (Fig. 3). Individual-level statistics had high sensitivity for both survey-reported and register-recorded coverage (Additional file 6).

### Objective 2: Denominator options for KMC coverage

Using an all LBW (≤2499 g) denominator option gave very similar results for survey-reported and register-recorded coverage compared to the ≤2000 g denominator results (Additional file 7).

### Objective 3: Gaps in coverage and quality of KMC and measurement

**Fig. 4:**
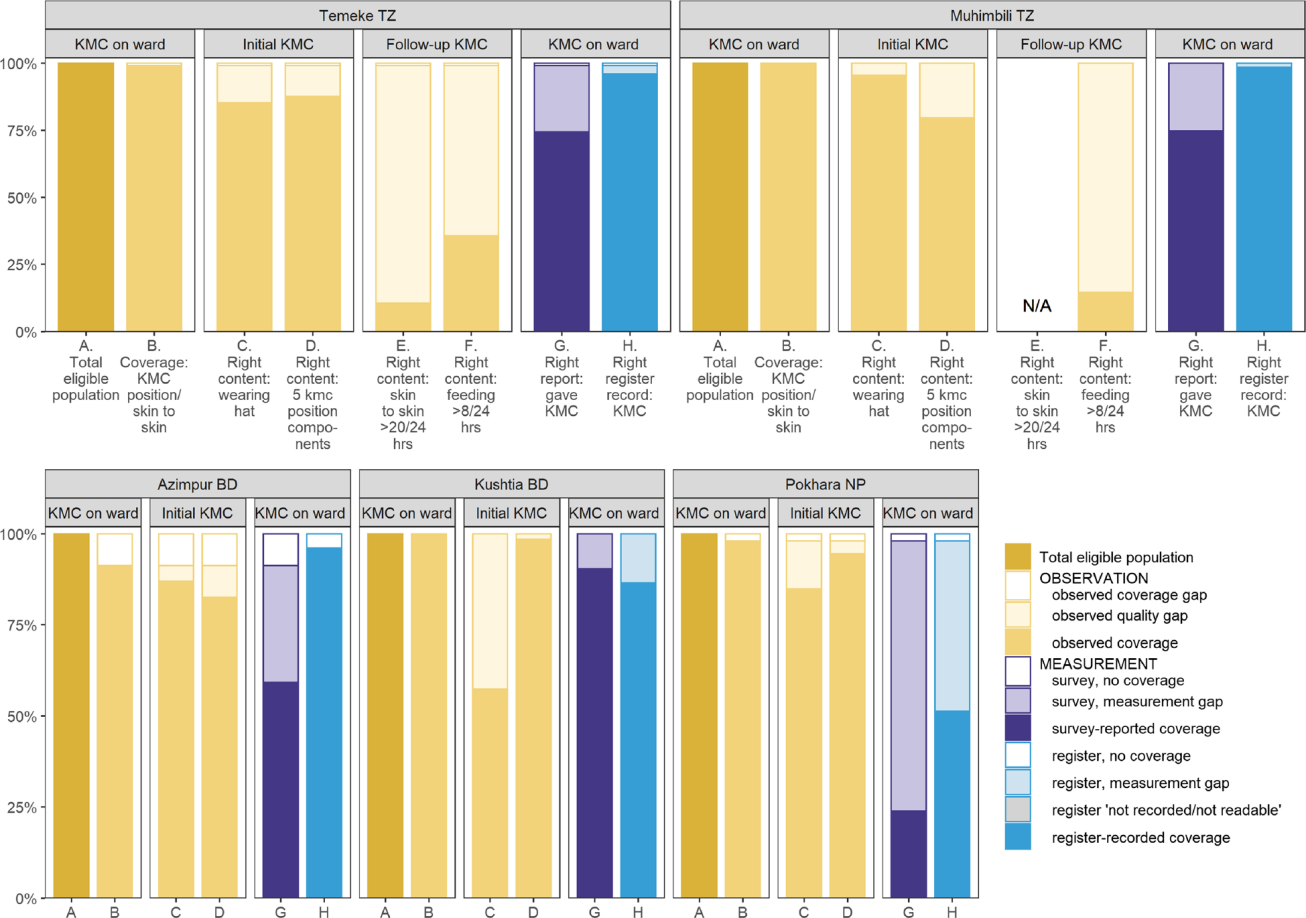
Gap analysis for KMC coverage, quality of care and measurement, EN-BIRTH study (*n* = 840). Among babies < 2000 g. Further details of content of care in Additional files 8 (≤2000 g) and 9 (all admissions). KMC daily dose in Additional file 12. KMC = kangaroo mother care, BD = Bangladesh, NP = Nepal, TZ = Tanzania

Figure 4 illustrates provision of care gap analyses for newborns stratified by hospital for (A) eligible admitted babies ≤2000 g, (B) KMC coverage (upright position/skin-to-skin (C) wearing a hat (D) all five position components. There were no substantial differences for babies ≤2499 g. Only 13.2% of mothers used a special KMC wrap, otherwise using a cloth/shawl to secure the baby in position. The coverage of key recommended components of KMC are presented in Additional files 8 and 9.

**Fig. 5:**
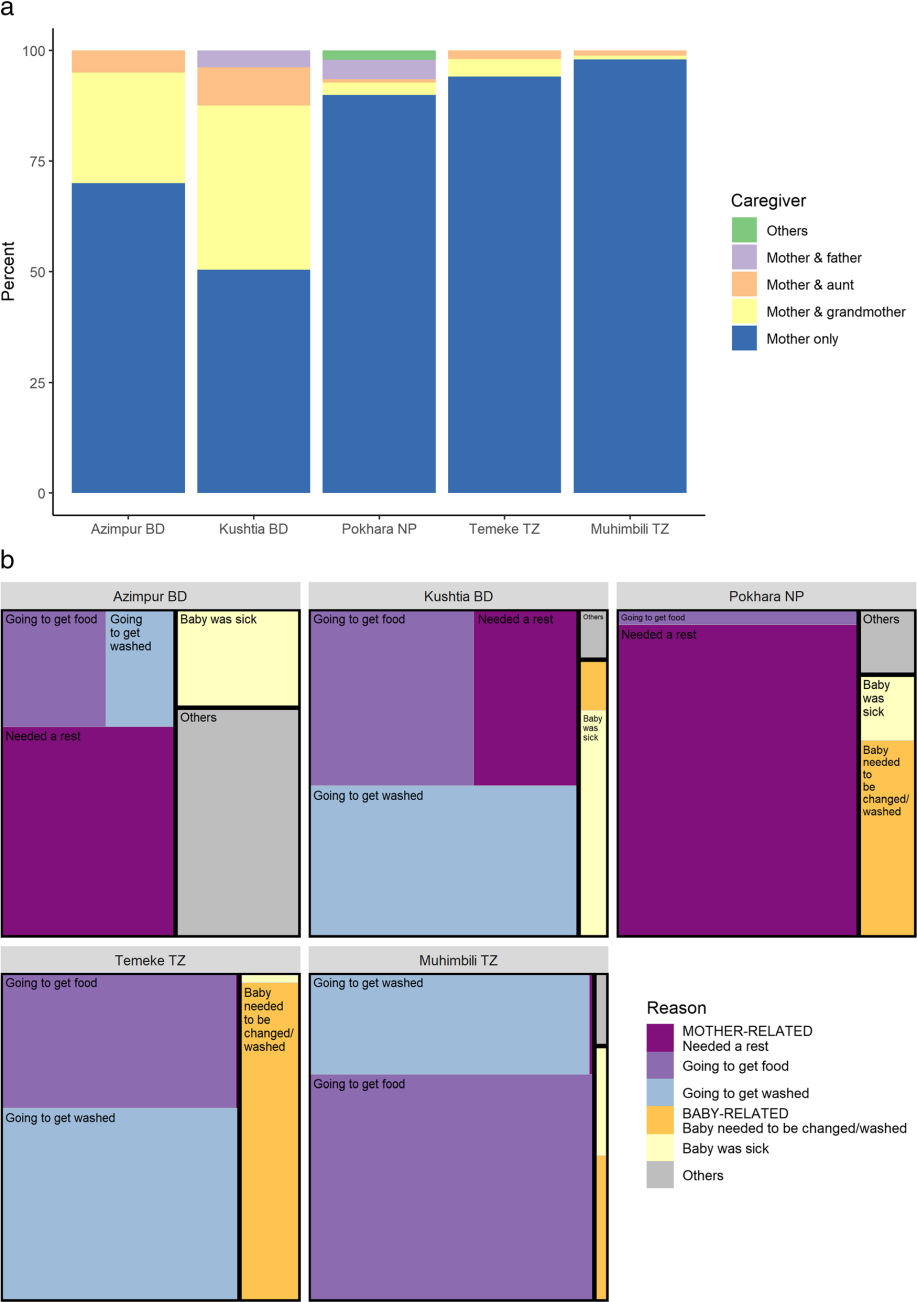
**a**: Observer-assessed supportive environment, EN-BIRTH study (n = 840). **b**: Survey-reported reasons preventing mothers from providing KMC, EN-BIRTH study (*n* = 652). BD = Bangladesh, NP = Nepal, TZ = Tanzania

Experience of KMC supportive environment results found mothers alone provided KMC 97.9% of the time in Muhimbili TZ and 50.5% in Kushtia BD, with the baby’s grandmother as the main family support (Fig. 5a). Survey report from 41.1% highlighted lack of ward arrangements to enable family support. Reasons preventing KMC during admission varied by site and were predominantly mother-related, including: needing to get food – highest in Muhimbili TZ (66.0%), needing a rest – highest in Pokhara NP (76.9%), and needing to wash – highest in Kushtia BD (41.7%) (Fig. 5b). Pre-discharge counselling was reported by 57.9%, topics included KMC position 24.7%, feeding practices 25.5% and need for follow-up visits 15%.

### Tanzanian hospitals only

#### “Right” content - KMC daily dose

Among target group ≤2000 g babies, KMC baby days with ≥20 point observations were available in Temeke (*n* = 6804). “Right” content, or ≥ 20 h of KMC skin-to-skin, was achieved for 10.6% of KMC baby days; 12–19 h by a further 75.4%; and < 12 h for 14.0%. Upright/vertical position and skin-to-skin both had a median time of 16 h (Fig. 4, column E, Additional file 10).

#### “Right” content – regular feeding

Feeding point observations ≥8/KMC baby day for mother-baby pairs ≤2000 g were 8212 in Temeke and 1352 in Muhimbili. Minimum or “Right feeding” frequency of ≥8 times/day was achieved on 35.6% KMC baby days in Temeke and 14.6% in Muhimbili. Observed mode of feeding for breastfeeding alone was higher in Temeke 17.7%, compared to 3.6% in Muhimbili. Assisted feeding was predominantly by cup, 31.9% of observed feeds were cup alone and a further 33.4% were both cup and breastfeeding. Mothers fed their babies > 99% of the time and NGTs were used for < 2% of feeds (Fig. 4, column F, Additional file 11, Additional file 12).

### Objective 4: Barriers and enablers to routine register recording

We conducted IDIs with 2–4 nurses working in KMC wards or KMC corners on neonatal wards in each hospital and EN-BIRTH study data collectors (*n* = 65) to reach saturation. One FGD was conducted in each hospital for triangulation (*n* = 5). Emerging themes specific for KMC registers around three process categories (Additional file 13) were:

#### Register design

Respondents explained that the KMC register is one of many documents to be completed, including patient case notes, monthly summary sheets, admission registers, ward round books, and discharge book. Health workers explained that readmission to KMC is not uncommon related to babies becoming unstable and register design currently does not accommodate this, affecting measurement:

*‘Today a child was admitted [again], she was under KMC [last month] but her condition went worse so she had to be shifted to neonatal ward, after some time that child got well and was shifted back to KMC this month. Now, I was asked what should be done, should they record her as a new admission, or she should continue with the previous one? I told them, no, the previous data has been already sent, so the child should be admitted afresh, in this month’.* - Health worker, Temeke TZ

#### Register filling

In all five hospitals, nurses took sole responsibility to document in registers. Documentation was described as overwhelming:

*‘From KMC, honestly, if you look at the proportion between documentation and care the one which is given first priority by nurses is care and then we forget to document. Because you find that there are many patients… ….and time is too short’*.- Health worker, Muhimbili TZ

*‘The main issue is manpower. Because of less people, there might be a chance of information being missed in documentation….if anything is missed during shift change that can hamper another shift’.* - Health worker, Pokhara NP

#### Register data use

Registers were valued in supporting patient care and were required for reporting and quality improvement.

*‘The treatments depend on the documentation e.g. the weight of the baby. Doctor provide the treatments based on the documentation. In my opinion, there is a strong relationship between the care and the documentation… Our works has no value without the documentation’.* - Health worker, Kushtia BD

*‘Record keeping helps us to provide quality services, it helps us to trace a patient who was discharged but she has come back, you get to see her previous issues which were documented’.* - Health worker, Temeke TZ

Despite many areas to document, health care workers reported that documentation is necessary.

*‘I think there are so many documents here in the ward because each document is important and supposed to be submitted somewhere’.* - Health worker, Muhimbili TZ

## Discussion

EN-BIRTH is the first study to assess validity of KMC coverage measurement compared to observation and explore dimensions of quality of care for a multi-country cohort in LMIC context. Admission to a KMC ward was an excellent marker of having received KMC, opening the way for tracking coverage from contact with KMC services. Data for action are urgently needed to accelerate scale-up of KMC for stable babies whilst research continues to establish whether unstable babies will also benefit [29].

Register data measurement of KMC coverage were accurate using specific KMC registers. However, register documentation in a non-specific column (in a general inpatient register for sick newborns) was incomplete in Pokhara NP. In the other four hospitals, using specific KMC registers, the high accuracy offers potential to link KMC register admission data to HMIS systems, including DHIS2. However, KMC registers are typically only located in KMC ward/corners, so tracking KMC coverage from these registers may underestimate intermittent KMC provided in other neonatal wards. This gap will be important to address if KMC for unstable newborns is introduced. Readmissions to KMC ward/corners could inflate KMC coverage and this needs further consideration. Defining the denominator for routine HMIS tracking will be critical, especially since LBW rate (≤2499 g) is already a core 100 health indicator but the KMC clinical need definition is currently ≤2000 g. Also the subtle definition differences of excluding babies weighing exactly 2500 g for LBW indicator, yet including those weighing 2000 g for KMC indicator adds a dimension of measurement complexity from aggregated routine data [30]. In Tanzania the national policy for KMC includes all LBW babies and in our study hospitals’ KMC wards we found 3% of our sample had admission weights > 2000 g. We collected KMC ward/corner admission weight for consistency, but notably mean age of admission varied by hospital, affecting the relationship between birth and admission weight. Register documentation was perceived by nurses as important, yet its priority competed against care for women and babies. Our findings may be generalizable to other similar settings where specific KMC registers are being implemented. However, our qualitative findings highlight the challenge of programme specific measurement adding to burden of duplication of data element documentation. Consideration to reduce any unnecessary duplication can enable health workers to efficiently use their time to support KMC mother-baby pairs as well as use the data they collect for quality improvement decisions e.g. increased frequency of feeding or daily dose of KMC.

Exit survey-report of KMC was also found to be highly accurate at the time of discharge from KMC ward/corners. Further evaluation to determine whether use in household survey is feasible should be considered. This could include measuring recall decay over the typical 2 to 5-year interval of population-based surveys, and also whether women who had not practiced KMC misreport having done so [31, 32]. Importantly, the household surveys’ sample size needs to be considered to ensure sufficient power to accurately measure KMC coverage for babies ≤2000 g [33]. These steps would facilitate assessment of inclusion of KMC indicators in household surveys such as DHS and MICS.

High-quality KMC, in both the dimensions of provision and experience of care is needed to have impact, but currently there is no consensus on definition. Prolonged skin-to-skin contact in KMC position is the cornerstone of KMC, although currently there is a lack of evidence for the optimal daily dose [6, 14, 15]. Wearing a hat is an important component of KMC for babies’ thermoregulation, especially in LMICs where ward temperatures are often unregulated. Yet families may not have access to appropriate sized hats for their preterm child. We found baby hat wearing coverage was lowest at 57.4% in Kushtia BD and highest 93.5% in Muhimbili, the site with the smallest babies and highest mean admission age, which may be related to hat availability after stabilisation in another ward. We suggest tracking of hat coverage may have potential for routine measurement as a tracer of content of care for these vulnerable infants. We found a median of 16 h in Temeke TZ, which was much higher than in an observational study conducted in Uganda [17]. Preterm babies require assisted feeding and we found a large quality gap. More than 8 feeds/day were only observed on 35.6% KMC baby days in Temeke TZ, and even lower 14.6% in Muhimbili TZ, despite the lowest mean admission weight of < 1300 g. Cup feeding was used frequently in both Tanzanian hospitals but NGT feeding rates were very low. The two Tanzanian study hospital KMC wards are different in layout which may affect quality of care: Temeke KMC ward is one room with every mother-baby pair visible from the nursing station compared to Muhimbili’s KMC ward over several rooms and an external nursing station.

The KMC mother-baby pair cohorts in the five study hospitals were notably different. Muhimbili TZ admitted smaller and older babies, after stabilisation on other neonatal wards, with longer KMC ward stays. As consensus is developed regarding components of high quality provision and experience of care for KMC mother-baby pairs, complexity of aggregate measurement of coverage and quality for diverse cohorts may need consideration. Disaggregating by admission weight may be complex due to regaining of weight that newborns lose immediately after birth. Birthweight may not be available for outborns or be heaped for inborns [30]. Individual longitudinal data linking KMC monitoring of outcome, nutrition and development is already a reality in the most established KMC national programmes [34].

Supportive KMC environment from health workers and family is crucial for the success of this continuous process of care, which may need to continue for weeks. Arrangements for other family members to be present during KMC admission is an important first step, but it was not common in these hospitals, which may improve if examples of supportive care is routinely measured [3, 6, 10, 17, 35].

### Strengths and limitations

The EN-BIRTH study is the first observational study to assess validity of measurement of KMC coverage. The qualitative data added insights into barriers and enablers to routine register recording. We established for the first time that in the LMIC context, contact with KMC services correlated well with receiving KMC. Our sample size of 840 mother-baby pairs from five hospitals in Bangladesh, Nepal and Tanzania enabled analyses on many dimensions of quality of care in the LMIC context. However, there are also limitations. The sample size varied across the study hospitals, with the lowest at 27 in Azimpur, which perhaps reflected lower levels of KMC implementation. We were unable to individually link observed KMC mother-baby pairs with target population stable babies ≤2000 g either born in the hospital labour ward or transferred from other neonatal wards, thus could not assess true denominator for coverage. Access for this population to KMC wards would be important to track for contact coverage. Frequency of KMC continuation observations was not consistent across all the study hospitals and only in the Tanzanian hospitals could hourly point observation data be analysed for feeding and in Temeke only for position. The differing KMC ward/corner layouts may have affected point observation comparisons, in a similar way that they might affect quality of care, an important consideration for the continuous practice of KMC. We were only able to interview 77% of the observed sample as women exited rapidly after discharge before the researchers could approach them, especially in the KMC corner of Pokhara NP, with mean length of stay only 1.5 days. Our study hospitals are all CEmONC hospitals, and the mothers in our sample had higher levels of education than national averages, so our findings might not be generalizable to measurement from KMC provided in other facilities. It is possible that the presence of researchers on the KMC wards/corners could have resulted in improved care or register documentation by health workers [25]. In Pokhara, the inter-rater reliability agreement for observation were unexpectedly low and might have affected validation results in that site. The more detailed analyses on daily dose of KMC and feeding were only from the Tanzanian hospitals, where KMC practice is more established compared to the Asian hospitals. We did not capture whether feeding was exclusively with breastmilk, which could be a dimension of quality for KMC. It was also beyond the scope of this study to explore how specific KMC implementation affected coverage and quality of KMC provision and experience.

### Research for improving measurement

Measurement of the process of KMC is complex and further research is needed. Tracking data from KMC wards/corners into HMIS has potential; implementation research is needed to understand data flow and quality, including efficient aggregation for the true denominator ≤2000 g. It is unlikely that all stable babies ≤2000 g have full access to KMC specific services, so interoperability between labour ward birthweight data and routine KMC data is an important area for research [14, 15]. To capture KMC coverage in the facility also requires including KMC provided on other wards, including special and intensive newborn care wards where babies are admitted for stabilisation before transition to KMC wards/corners. Moreover, exploring how to best measure population coverage for facility KMC as both inborn and outborn babies are admitted for KMC needs consideration. Measuring quality of the provision of KMC (daily dose, feeding, weight gain etc.), and the experience of care is unlikely to be feasible in routine registers or population-based surveys. Research is needed to explore other approaches, including case audits and special studies, with similar definitions across sites so comparisons can be made. Measurement research for standardised indicators of long-term health and well-being to maximise developmental and nutritional outcomes for KMC survivors is a key research priority [36]. Innovation regarding measurement of a KMC supportive environment – including appropriate physical space, health worker experience of care, and supportive supervision – is needed.

## Conclusions

Scale-up of KMC is a priority intervention and our results show that coverage of KMC could be tracked in routine systems by using count data on admission to KMC wards/corners, best measured with a specific KMC ward register. Further work is needed to understand if KMC can be tracked by household surveys, especially while coverage is low. Clear, measurable definitions of high quality KMC are needed for maximal impact of this intervention – with huge potential to improve outcomes for vulnerable newborns to survive and thrive.

## Supplementary Information

**Additional file 1.** STROBE checklist.

**Additional file 2.** Metadata definitions of selected indicators for validity testing, EN-BIRTH study.

**Additional file 3.** Background characteristics of women observed in KMC ward, EN-BIRTH study.

**Additional file 4.** Routine register design in 5 EN-BIRTH study hospitals and data quality dimensions.

**Additional file 5.** Data quality assurance for gold standard – KMC Double Observation and Data Entry, EN-BIRTH study.

**Additional file 6.** Individual-level validation in exit survey report and register record of KMC indicator, EN-BIRTH study, (*n* = 840).

**Additional file 7.** Comparison of KMC denominator options - coverage observed, survey-reported, register-recorded coverage, EN-BIRTH study, observed *n* = 840.

**Additional file 8.** Markers for quality of KMC by hospital, EN-BIRTH study ≤2000 g, *n* = 815 observed.

**Additional file 9.** Markers for quality of KMC by hospital, EN-BIRTH study all weights, n = 840 observed.

**Additional file 10.** Box plots KMC daily dose: upright/vertical position, skin-to-skin, EN-BIRTH study Temeke Hospital, Tanzania (*n* = 6804 point observations).

**Additional file 11.** Observed feeding practices for KMC mother-baby pairs, EN-BIRTH study Tanzania sites (*n* = 22,793 point observations).

**Additional file 12.** Flow diagram for analyses of KMC continuity – Tanzania sites, ≤2000 g EN-BIRTH study.

**Additional file 13.** Barriers and enablers to routine reporting and documentation for KMC in the EN-BIRTH study.

**Additional file 14.** Ethical approval of local institutional review boards, EN-BIRTH study.

## Data Availability

The datasets generated during and/or analysed during the current study are available on LSHTM Data Compass repository, https://datacompass.lshtm.ac.uk/955/.

## Abbreviations

BD: Bangladesh
CEmONC: Comprehensive Emergency Obstetric and Newborn care
CIFF: Children’s Investment Fund Foundation
DHS: The Demographic and Health Surveys Program
DHIS2: District Health Information Software 2
ENAP: *Every Newborn* Action Plan now branded as *Every Newborn*
EN-BIRTH: *Every Newborn*-Birth Indicators Research Tracking in Hospitals study
FGD: Focus group discussion
g: grammes
HMIS: Health Management Information Systems
icddr,b: International Centre for Diarrheal Disease Research, Bangladesh
IDI: in-depth interview
IHI: Ifakara Health Institute
KMC: Kangaroo mother care
LMIC: Low- and middle-income country
LSHTM: London School of Hygiene & Tropical Medicine
MARCH: Centre for Maternal, Adolescent, Reproductive & Child Health, LSHTM
MCHTI: Maternal and Child Health Training Institute, Azimpur, Bangladesh
MUHAS: Muhimbili University of Health and Allied Sciences
MICS: Multiple Indicator Cluster Survey
NGT: nasogastric tube
NP: Nepal
PRISM: Performance of Routine Information System Management
TZ: Tanzania
UNICEF: United Nations Children’s Fund
WHO: World Health Organization
