# Supplementary material for: Kangaroo mother care: EN-BIRTH multi-country validation study
Source: BMC Pregnancy Childbirth. 2021 Mar 26;21(Suppl 1):231. doi: 10.1186/s12884-020-03423-8 (PMC7995571; doi:10.1186/s12884-020-03423-8)
Supplement: Supplementary file 2 — Additional file 2. Metadata definitions of selected indicators for validity testing, EN-BIRTH study. [file 12884_2020_3423_MOESM2_ESM.pdf]

Every Newborn BIRTH multi-country validation study: informing measurement of coverage and quality of maternal and newborn care

Kangaroo mother care: EN-BIRTH multi-country validation study

Additional file 2: Metadata definitions of selected indicators for validity testing, EN-BIRTH study

Indicator	Numerator		Denominator used for analyses in this paper
**Kangaroo Mother Care**	Observed to:	Receive Kangaroo Mother Care during admission (KMC position and/or Skin-To-Skin care)	All babies observed on KMC ward/corner
	Survey maternal report, answered yes to:	"Was your baby born early/ Baby born before the expected date of delivery /born too soon or too small and had extra care"<br>and "Did your baby receive Kangaroo Mother Care?"	
	Register Data Extraction, documented:	In Kangaroo Mother Care register	

Observation compared to women's exit survey report or register, data extraction
