# Supplementary material for: Kangaroo mother care: EN-BIRTH multi-country validation study
Source: BMC Pregnancy Childbirth. 2021 Mar 26;21(Suppl 1):231. doi: 10.1186/s12884-020-03423-8 (PMC7995571; doi:10.1186/s12884-020-03423-8)
Supplement: Supplementary file 4 — Additional file 4. Routine register design in 5 EN-BIRTH study hospitals and data quality dimensions. [file 12884_2020_3423_MOESM4_ESM.pdf]

Every Newborn BIRTH multi-country validation study: informing measurement of coverage and quality of maternal and newborn care

Kangaroo mother care: EN-BIRTH multi-country validation study

Additional file 4: Routine register design in five EN-BIRTH study hospitals and data quality dimensions

		Bangladesh		Nepal	Tanzania	
		**Azimpur Tertiary**	**Kushtia District**	**Pokhara Regional**	**Temeke Regional**	**Muhimbili National**
**Kangaroo Mother Care**						
**Register design:**		specific KMC register	specific KMC register	non-specific newborn	specific KMC register	specific KMC register
Column heading		23 specific columns	23 specific columns	KMC partial or continuous	38 specific columns	38 specific columns
Data element completed if KMC initiated		entered in register	entered in register	tick	entered in register	entered in register
Data element completed if KMC not initiated		not entered in register	not entered in register	blank	not entered in register	not entered in register
**Completeness**	Data element recorded in register	100.0%	98.5%	93.0%	99.1%	98.8%
**External Consistency**						
Indicator:	Observed coverage %	100.0%	99.9%	99.9%	99.8%	99.5%
Indicator:	Measured coverage - register recorded %	100.0%	97.8%	21.2%	84.8%	85.2%
Measurement gap	Register recorded and observed	0.0%	2.1%	78.7% underestimate	15.0% underestimate	14.3% underestimate

Key

no column for data element
non-specific column for data element
specific column

>20%	Poor
16-20%	Moderate
11-15%	Good
6-10%	Very Good
0-5%	Excellent

Register design: specific column, non-specific column, no column
Data quality dimensions: completeness and external consistency compared to gold standard observation
Heat mapped showing cut-offs
