# Supplementary material for: Kangaroo mother care: EN-BIRTH multi-country validation study
Source: BMC Pregnancy Childbirth. 2021 Mar 26;21(Suppl 1):231. doi: 10.1186/s12884-020-03423-8 (PMC7995571; doi:10.1186/s12884-020-03423-8)

Every Newborn BIRTH multi-country validation study: informing measurement of coverage and quality of maternal and newborn care

Kangaroo mother care: EN-BIRTH multi-country validation study

Additional File 10: Box plots KMC daily dose: upright/vertical position, skin-to-skin, EN-BIRTH study Temeke Hospital, Tanzania (n=6,804 point observations)

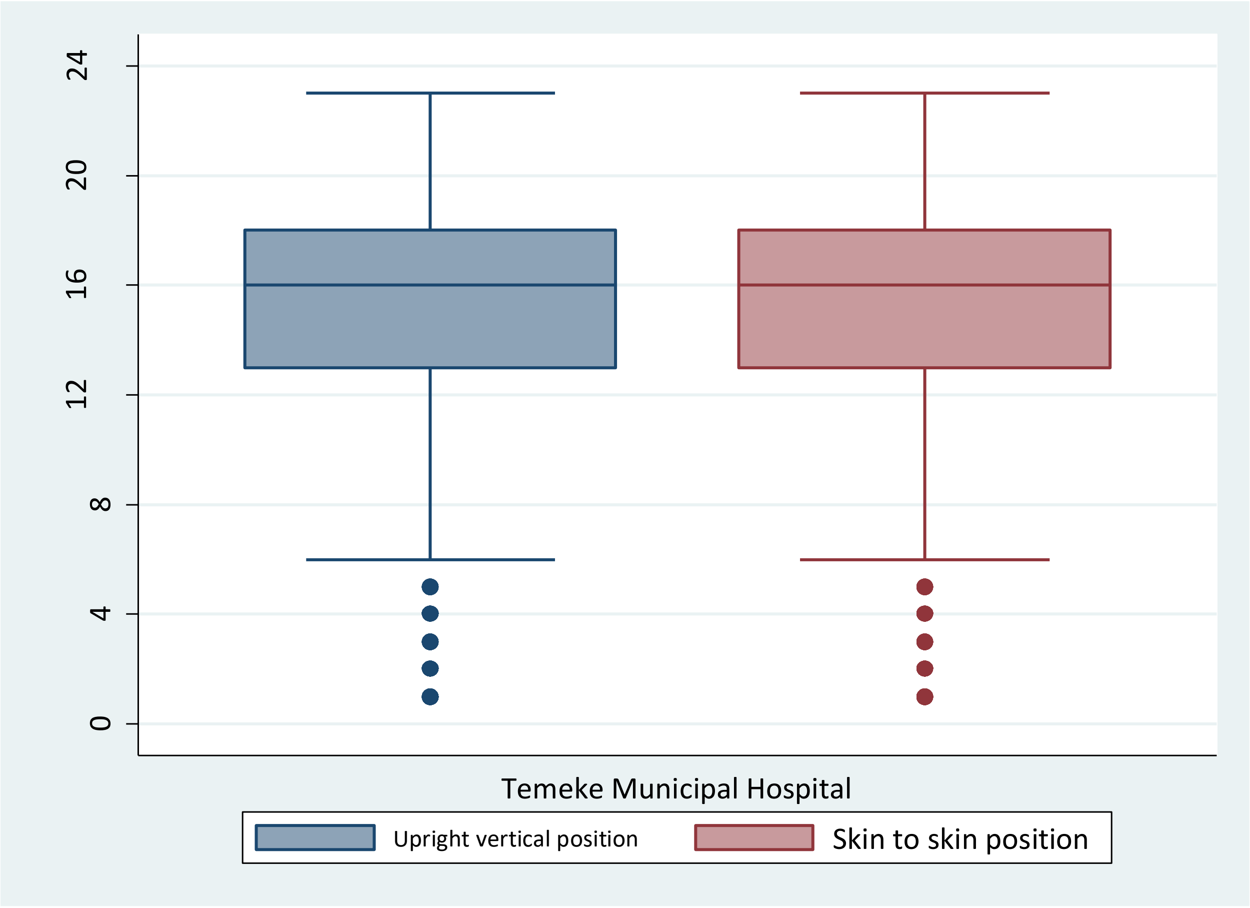

Supplement: Supplementary file 10 — Additional file 10. Box plots KMC daily dose: upright/vertical position, skin-to-skin, EN-BIRTH study Temeke Hospital, Tanzania (n = 6804 point observations). [file 12884_2020_3423_MOESM10_ESM.pdf]
