# Supplementary material for: Kangaroo mother care: EN-BIRTH multi-country validation study
Source: BMC Pregnancy Childbirth. 2021 Mar 26;21(Suppl 1):231. doi: 10.1186/s12884-020-03423-8 (PMC7995571; doi:10.1186/s12884-020-03423-8)

Every Newborn BIRTH multi-country validation study: informing measurement of coverage and quality of maternal and newborn care

Kangaroo mother care: EN-BIRTH multi-country validation study

Additional File 11: Observed feeding practices for KMC mother-baby pairs, EN-BIRTH study Tanzania sites (n=22,793 point observations)

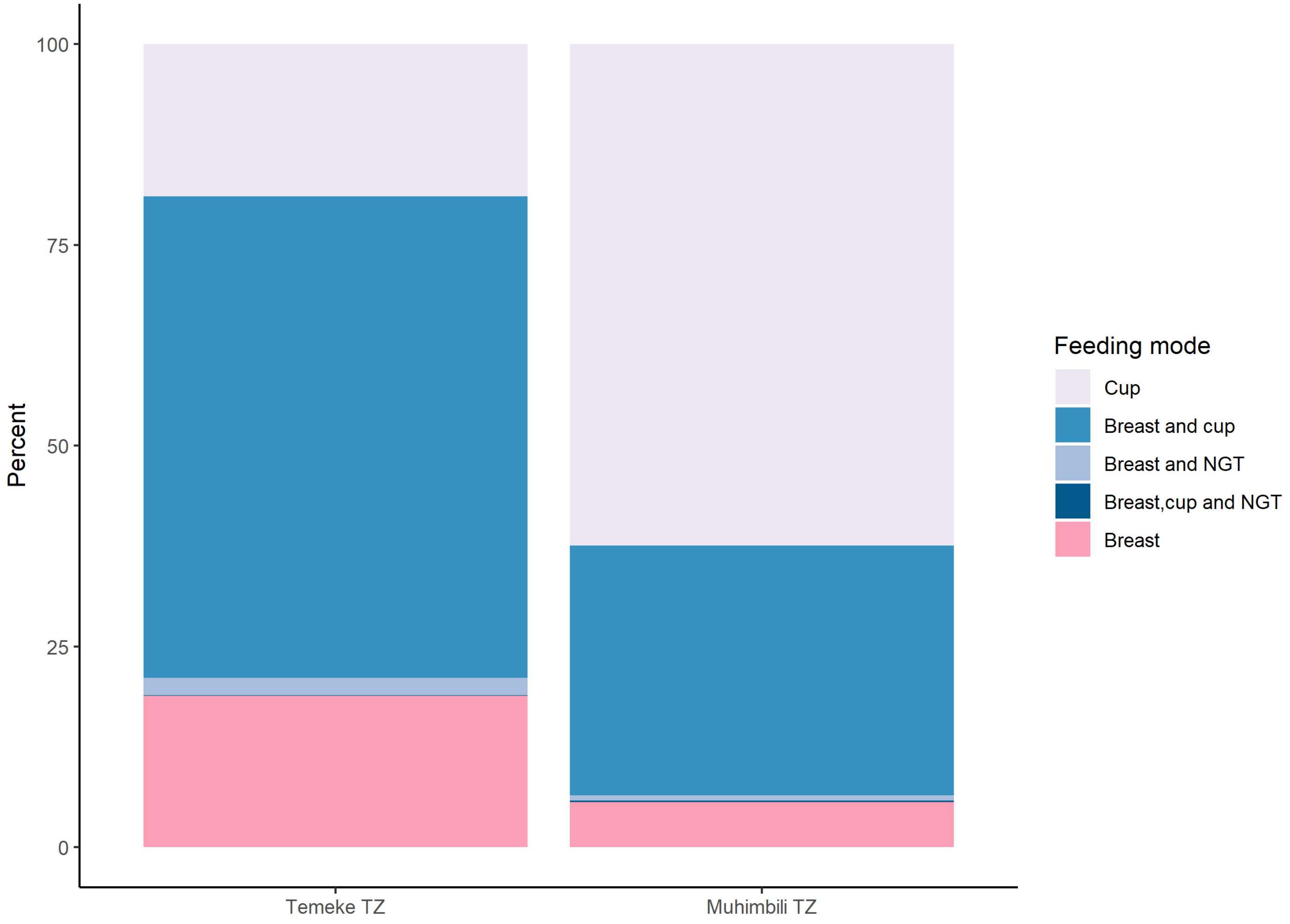

Supplement: Supplementary file 11 — Additional file 11. Observed feeding practices for KMC mother-baby pairs, EN-BIRTH study Tanzania sites (n = 22,793 point observations). [file 12884_2020_3423_MOESM11_ESM.pdf]
