# Supplementary material for: Kangaroo mother care: EN-BIRTH multi-country validation study
Source: BMC Pregnancy Childbirth. 2021 Mar 26;21(Suppl 1):231. doi: 10.1186/s12884-020-03423-8 (PMC7995571; doi:10.1186/s12884-020-03423-8)

Every Newborn BIRTH multi-country validation study: informing measurement of coverage and quality of maternal and newborn care

Kangaroo mother care: EN-BIRTH multi-country validation study

Additional file 12: Flow diagram for analyses of KMC continuity – Tanzania sites, ≤2000g EN-BIRTH study

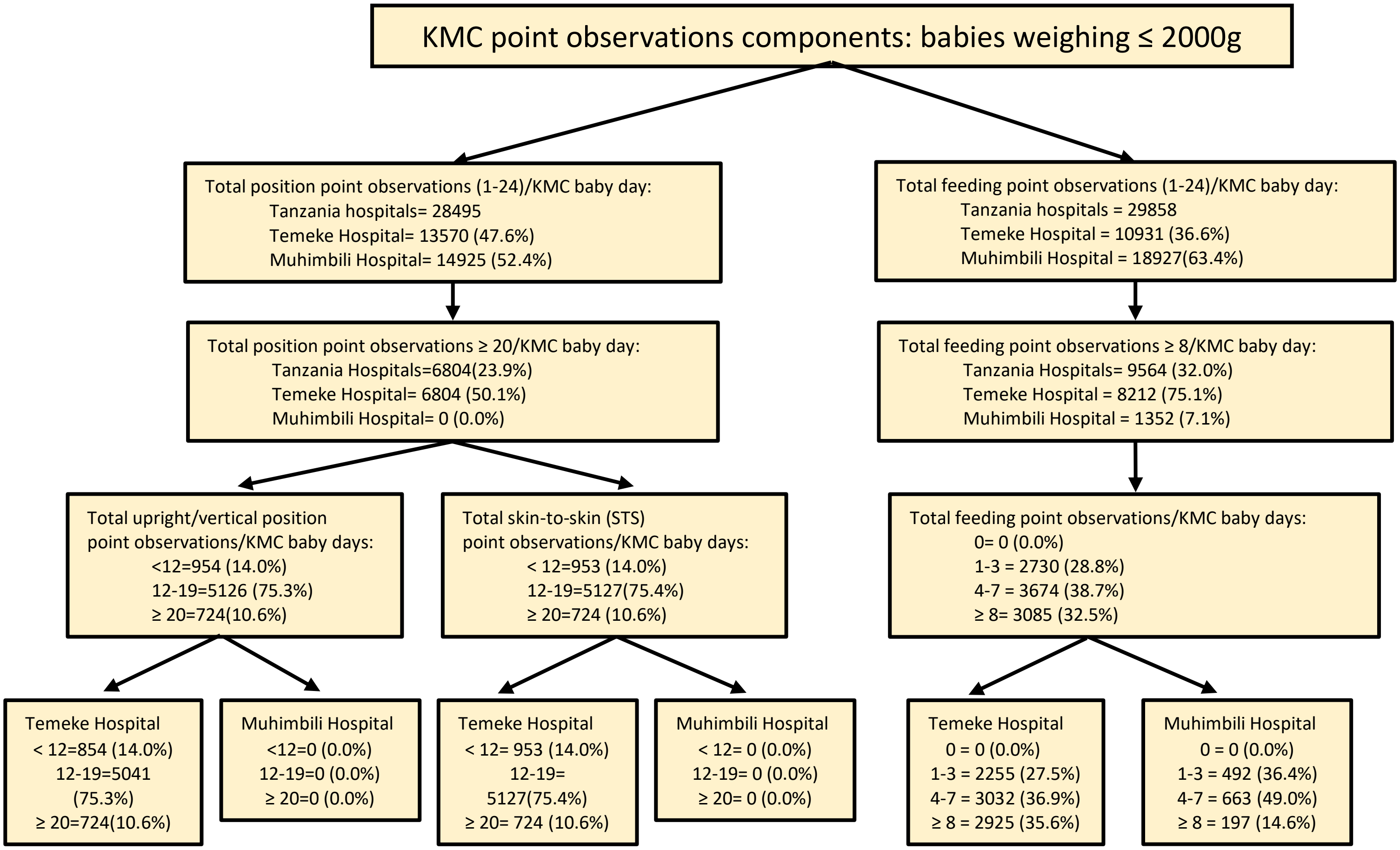

Supplement: Supplementary file 12 — Additional file 12. Flow diagram for analyses of KMC continuity – Tanzania sites, ≤2000 g EN-BIRTH study. [file 12884_2020_3423_MOESM12_ESM.pdf]
