# Supplementary material for: Kangaroo mother care: EN-BIRTH multi-country validation study
Source: BMC Pregnancy Childbirth. 2021 Mar 26;21(Suppl 1):231. doi: 10.1186/s12884-020-03423-8 (PMC7995571; doi:10.1186/s12884-020-03423-8)

Every Newborn BIRTH multi-country validation study: informing measurement of coverage and quality of maternal and newborn care

Kangaroo mother care: EN-BIRTH multi-country validation study

Additional File 13: Barriers and Enablers to Routine Reporting and Documentation for KMC in the EN-BIRTH study

Enablers

Factors that promote accurate register completion

Barriers

Factors that limit the use or accuracy of register completion

Data culture

Shared values related to information systems

Categories

Key categories identified acting as barriers and enablers to routine documentation: register design, register filling and register use

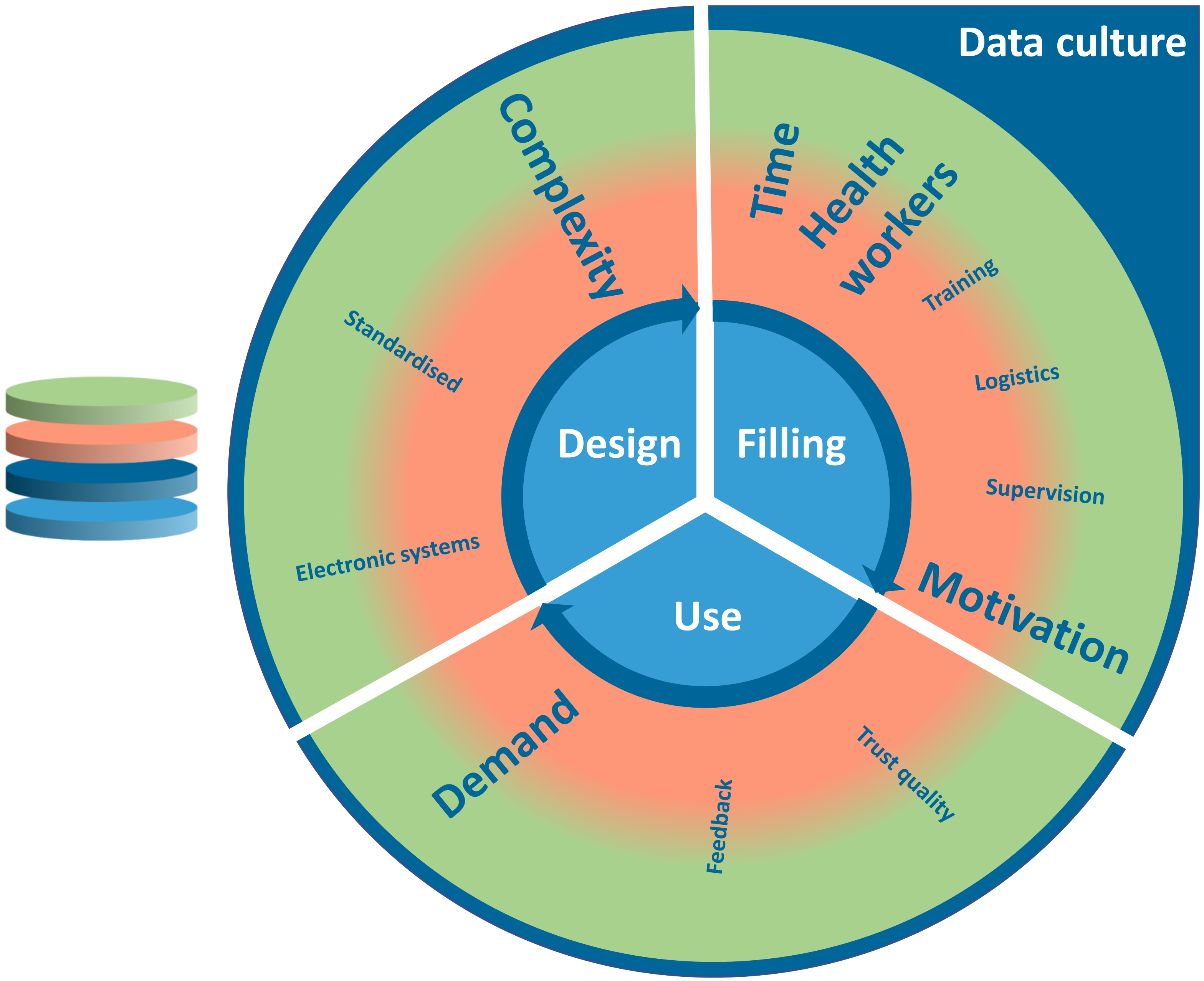

Supplement: Supplementary file 13 — Additional file 13. Barriers and enablers to routine reporting and documentation for KMC in the EN-BIRTH study. [file 12884_2020_3423_MOESM13_ESM.pdf]
